# The association between automatic thoughts about eating, the actual–ideal weight discrepancies, and eating disorders symptoms: a longitudinal study in late adolescence

**DOI:** 10.1007/s40519-014-0099-2

**Published:** 2014-02-09

**Authors:** Karolina Zarychta, Aleksandra Luszczynska, Urte Scholz

**Affiliations:** 1 University of Social Sciences and Humanities, 30b Ostrowskiego Street, 53-238 Wrocław, Poland; 3 University of Zurich, Binzmühlestr. 14, Box 14, 8050 Zurich, Switzerland; 4 Trauma, Health, and Hazards Center, University of Colorado at Colorado Springs, 1861 Austin Bluffs Pkwy, Colorado Springs, CO 80918 USA

**Keywords:** Eating disorders, Eating disorders symptoms, Automatic thoughts about eating, Actual–ideal weight discrepancy

## Abstract

**Objective:**

This study tested the reciprocal relationships between automatic thoughts about eating and the actual–ideal weight discrepancies, and their role in the formation and maintenance of eating disorders (ED) symptoms in a non-clinical sample of adolescents. In particular, we investigated whether thoughts about eating mediated the effects of weight discrepancies on ED formation and whether weight discrepancies mediated the effects of thoughts about eating on ED formation were investigated.

**Method:**

Data were collected three times, with a 2-month interval between Time 1 (T1) and Time 2 (T2), and a 9-month interval between T2 and Time 3 (T3). Adolescents (*N* = 55) aged 15–18 filled out the SCOFF Questionnaire, assessing eating disorders symptoms, and the Eating Disorder Thoughts Questionnaire, evaluating automatic thoughts. To assess weight discrepancies questions about actual (subjectively reported) and ideal body weight were asked followed by objective measurement of height and weight.

**Results:**

Negative thoughts about eating (T2) mediated the relation between weight discrepancies (T1) and symptoms of anorexia and bulimia (T3). In addition, the association between negative thoughts (T1) and eating disorders symptoms (T3) was mediated by weight discrepancies (T2).

**Conclusion:**

The negative thoughts and the actual (both subjectively reported and objectively measured)–ideal weight discrepancies constitute a vicious cycle, related to higher ED symptoms. Prevention of eating disorders should be directed to adolescents who manifest large weight discrepancies or high levels of negative thoughts about eating, as they are at risk for developing eating disorder symptoms.

## Introduction

Recent research on eating disorders (ED) etiology and maintenance suggests that cognitive factors play the key role for symptom formation. Cognitive models of ED [1–4] focus primarily on the way people perceive their bodies, the contents of their thoughts, perceptions of body weights and shapes. Individuals who are preoccupied with weight and body shape let their outer appearance affect their self-evaluations. This may lead to excessive concentration on weight loss and self-control and to pathological behaviors (i.e. restrictive diet, compulsive exercise, abuse of laxatives, or self-induced vomiting). People diagnosed with ED present the largest distortion in body perceptions [5].

Besides perceptions of body weight and shape, automatic thought processes are among key determinants of ED. According to one of the key cognitive ED models, proposed by Cooper [1, 6], there are three types of automatic thoughts about eating (positive, negative, and permissive). These thoughts may act in a vicious circle, prompting disturbed eating behaviors. Positive thoughts about eating are associated with the perceived benefits of eating (e.g. “If I eat it will all hurt less inside”). Negative thoughts about eating refer to the perceived consequences (e.g. “My clothes won’t fit anymore”). Permissive thoughts make it easy to start and keep on eating (e.g. “I’ll just have a little bit more”). Originally, Cooper’s [1, 6] model was used to explain symptoms of bulimia, but recently it has been proposed that core psychopathology across ED seems similar and it is initiated/maintained by the same cognitions [7]. Further, empirical evidence confirms similar patterns of associations between the three types of automatic thoughts and both bulimia and anorexia [2].

Bulimic episodes are triggered by negative thoughts about oneself, which relate particularly to body weight and shape, and are connected to low self-esteem, sense of shame, guilt and failure, as well as social isolation and depression symptoms [8]. Among individuals with ED symptoms food intake frequently ends with negative thoughts about eating, which consequently lead to the feeling of being fat and negative thoughts about oneself [1, 3]. Negative thoughts include statements referring to control over eating and losing control [2] which are present in bulimia and anorexia [2].

In the case of anorexia, the cognitive model emphasizes the role of positive and negative thoughts, with negative thoughts about oneself playing the key role for ED symptoms formation [1, 2]. Positive thoughts in anorexia may for example help maintain failure to achieve an adequate food intake and contribute to the “drive for thinness” [2]. In case of bulimia, eating episodes are accompanied by positive thoughts, which temporarily turn the attention away from unpleasant emotions and beliefs. Negative thoughts are activated immediately afterwards and the fear of consequences appears. Dissonance between the positive and negative thoughts brings about distress and further formation of permissive thoughts. Once these thoughts are activated, more eating takes place, potentially resulting in binge eating episodes [2]. Negative thoughts about oneself reappear after these binge eating episodes, eventually resulting in fasting, purging, dieting and vomiting [1, 2].

Cognitive models of ED assume that evaluations of body weight and discrepancies between actual and perceived weight are key factors in the development and maintenance of anorexia and bulimia [7]. The cognitive model of ED, proposed by Fairburn [7, 9], indicates that low self-esteem, mood intolerance, clinical perfectionism, and interpersonal difficulties are among the mechanisms responsible for maintenance of ED symptoms. Those factors, however, operate only in certain patients with anorexia or bulimia [7, 9], whereas the common core mechanism includes body weight over-evaluations, which are assumed to affect ED symptoms across different kinds of eating disorders [7].

The self-discrepancy theory suggests that individuals evaluate themselves in the context of who they currently are, who they wish to be, and who they should be [10]. Therefore, three components of the self may be distinguished: (1) actual-self, (2) ideal-self, and (3) ought-self. Discrepancies between them may increase the vulnerability to negative emotional states. Thus, individuals tend to reduce arising discrepancies. Self-discrepancy theory can be adopted to explaining effects of body weight and shape evaluations on ED symptoms formation [10–13]. As far as ED are concerned, the most striking discrepancies may occur between the actual-self and ideal-self (in bulimia), and the actual-self and ought-self (in anorexia) [13]. Since individuals with both anorexia and bulimia symptoms recognize their body weight as the central part of their self [14], it is assumed that the biggest discrepancies appear among their actual, ideal and ought weight.

The theoretical framework for the present study refers to the cognitive model of ED proposed by Cooper et al. [1], which emphasizes the role of the automatic thoughts in the formation and maintenance of ED symptoms, and the self-discrepancy theory [10] in relation to the weight discrepancies observable among people affected by ED. We account for such predictors as negative, positive, and permissive thoughts, the actual (subjectively reported and objectively measured)–ideal weight discrepancies, with the ED symptoms constituting the main outcome variable. Using a prospective design we aimed at examining the associations between these cognitive predictors in a non-clinical sample of adolescents who are the most vulnerable age group to the occurrence of ED symptoms.

There is no sufficient empirical evidence on predictive or mediating effects of the above-outlined variables (thoughts about eating and weight discrepancies) on one another. Therefore, the present study aimed at analyzing possible reciprocal relations. First, it was hypothesized that the automatic thoughts would mediate the relationship between weight discrepancies and ED symptoms. Second, it was hypothesized that the association between the automatic thoughts and ED symptoms would be mediated by weight discrepancies.

## Materials and methods

### Participants

At Time 1 (T1) 55 adolescents (65.5 % were girls) participated in the study. They were 16- to 18-year old (*M* = 16.24, SD = 0.61). Fifty-two individuals (92.86 %) took part in the study at Time 2 (T2, 2 months later) and 47 individuals (83.93 %) provided their data at Time 3 (T3, 9 months later).

The study was conducted during school hours. Individuals were informed about the objectives and the procedure of the study. At T1 each participant voluntarily filled in a questionnaire. Afterwards, participants moved individually to another room, where their height and weight were measured. The procedure was repeated at T2 and T3. Experimenters were available for consultations after the study completion. All participants provided their informed consent. The study was approved by the Institutional Review Board.

### Materials

Mean, standard deviations and reliability coefficients are presented in Table 1.

**Table 1:** Descriptive statistics, reliability, and correlations between the study variables at T1, T2 and T3

		*M* (SD)	*α*	2	3	4	5	6	7	8	9	10	11	12	13	14	15	16	17	18
1	T1 ED symptoms	0.72 (1.02)		0.88***	0.68***	0.64***	0.72***	0.32**	0.23^†^	0.36**	0.20^†^	−0.09	0.01	<0.01	0.42***	0.40***	0.41***	0.44***	0.37**	0.35***
2	T2 ED symptoms	0.67 (1.23)			0.73***	0.52***	0.69***	0.22^†^	0.17	0.37**	0.14	−0.09	0.02	−0.04	0.37**	0.43***	0.35**	0.40***	0.37**	0.26*
3	T3 ED symptoms	0.70 (1.12)				0.50***	0.60***	0.63***	0.16	0.35**	0.56***	−0.02	0.22^†^	0.28*	0.29*	0.32**	0.33**	0.29*	0.27*	0.24*
4	T1 negative thoughts	26.31 (22.88)	0.90				0.83***	0.57***	0.57***	0.56***	0.37**	0.35**	0.36**	0.30**	0.46***	0.42***	0.47***	0.48***	0.43***	0.48***
5	T2 negative thoughts	20.61 (20.77)	0.88					0.54***	0.54***	0.68***	0.40***	0.17	0.30*	0.25*	0.44***	0.41***	0.42***	0.45***	0.39***	0.37**
6	T3 negative thoughts	22.72 (23.18)	0.92						0.17	0.22^†^	0.53***	0.11	0.23^†^	0.47***	0.43***	0.39***	0.49***	0.39***	0.39***	0.50***
7	T1 positive thoughts	14.62 (17.25)	0.84							0.77***	0.50***	0.49***	0.48***	0.30*	0.10	0.09	0.18	0.15	0.13	−0.01
8	T2 positive thoughts	10.76 (15.12)	0.83								0.67***	0.37**	0.50***	0.29*	0.09	0.06	0.13	0.14	0.07	<0.01
9	T3 positive thoughts	10.85 (15.64)	0.81									0.29*	0.47***	0.57***	0.05	−0.03	0.11	0.06	<−0.01	−0.01
10	T1 permissive thoughts	31.32 (23.44)	0.65										0.75***	0.57***	−0.16	−0.12	−0.04	−0.14	−0.11	0.02
11	T2 permissive thoughts	24.23 (21.47)	0.68											0.79***	0.04	<0.01	0.03	0.05	−0.01	0.06
12	T3 permissive thoughts	23.08 (20.51)	0.72												0.06	−0.01	0.09	0.05	0.02	0.11
13	T1 SI weight discrepancy	3.47 (10.66)														0.92***	0.87***	0.99***	0.95***	0.89***
14	T2 SI weight discrepancy	3.21 (9.23)															0.88***	0.91***	0.87***	0.85***
15	T3 SI weight discrepancy	2.98 (8.51)																0.86***	0.89***	0.87***
16	T1 OI weight discrepancy	3.87 (10.65)																	0.94***	0.88***
17	T2 OI weight discrepancy	3.00 (9.90)																		0.87***
18	T3 OI weight discrepancy	3.54 (9.58)																		

*** *p* < 0.001, ** *p* < 0.01, * *p* < 0.05, ^†^ *p* < 0.1

T1, Time 1; T2, Time 2; T3, Time 3; ED, eating disorders; SI weight discrepancy, actual (subjectively reported)–ideal weight discrepancy; OI weight discrepancy, actual (objectively measured)–ideal weight discrepancy

#### Eating disorder thoughts questionnaire

Three types of automatic thoughts (positive, negative, and permissive) were measured with the Eating Disorder Thoughts Questionnaire [2], translated into Polish by the study author using the decentering technique [15]. The scale consists of 26 items, rated on a scale ranging from 0 (“I do not usually believe this at all”) to 100 (“I am usually completely convinced that this is true”).

#### Symptoms of eating disorders

The SCOFF Questionnaire, developed by Morgan et al. [16], was used as a screening tool for identifying ED symptoms in this non-clinical group. The name of the questionnaire is derived from the first letters of the keywords included in each of the five SCOFF items: sick, control, one stone, fat and food. Translation to Polish was conducted using decentering technique [16]. Responses are given on a dichotomous scale (Yes 1 or No 0), with a total score ≥2 indicating a likely case of ED.

#### Assessment of discrepancies between the actual and ideal weight

To obtain the actual weight (subjectively reported) of the respondents, they were asked to provide their weight in kilograms. In order to measure the ideal weight, participants were asked to provide answers to a following question: “How much would you like to weight?” The actual body weight (objectively measured) was assessed using a certified body weight scale (Beurer BF 25). The actual (subjectively reported)–ideal weight discrepancy was calculated by subtracting the actual weight (subjectively reported) from the ideal weight, while the actual (objectively measured)–ideal weight discrepancy was calculated by subtracting the actual weight (objectively measured) from the ideal weight.

### Data Analysis

Preliminary analysis indicated that the data were missing completely at random (*p* > 0.05 for Little’s *χ* ^2^); therefore, missing data (including dropout cases data) were replaced using the multiple imputation method [17]. Positive (T1, T2, T3), negative (T1, T2, T3), and permissive (T1, T2, T3) thoughts, ED symptoms (T1, T2, T3), the actual (subjectively reported)–ideal weight discrepancy (T1, T2, T3), and the actual (objectively measured)–ideal weight discrepancy (T1, T2, T3) were included in the regression method for multiple imputation. As suggested by MacKinnon [18], the independent variable, the mediator, and the dependent variable in the respective equations were measured at three different time points (T1, T2, and T3) to establish temporal precedence.

The mediation hypotheses were tested with the INDIRECT macros by Hayes [19], which applies regression analysis to test the associations among the independent variable, the mediator, and the dependent variable. INDIRECT allows for testing the significance of indirect effects and for testing the effects of multiple mediators, operating in a parallel manner [20]. Further, INDIRECT uses the bootstrapping approach to assess the size and significance of indirect effects. Bootstrapping used to inferring indirect effects is recommended in particular in smaller and non-normally distributed samples, in contrast to the normal theory approach of mediation [20]. Bootstrapping-based mediation analysis offers an alternative to mediation analysis with latent variables in structural equation modeling programs, in particular when the sample size is small in relation to the number of parameters in the equation [19].

## Results

### Dropout analysis

Completers did not differ from those who dropped out at T2 and/or T3 in terms of weight (ideal, subjectively reported, objectively measured), height (ideal, subjectively reported, objectively measured), body fat, BMI, the actual (subjectively reported and objectively measured)–ideal weight discrepancies, negative, and permissive thoughts, all *F* < 2.19, *p* > 0.10, or gender, *χ* ^2^ (1) = 1.22, *p* = 0.27. Dropouts and completers differed slightly in terms of positive thoughts, *F* (1, 52) = 4.17, *p* = 0.046 with dropouts reporting more positive thoughts (*M* = 19.33, SD = 14.59) than completers (*M* = 12.40, SD = 9.56). There was no effect of dropping out on ED symptoms, *F* (1, 52) = 1.92, *p* = 0.173.

As gender, BMI or body fat may constitute relevant confounders in predicting ED symptoms, the associations between these variables measured at T1 and symptoms at T3 were tested. The relationships turned out to be negligible (*p*_s_ > 0.053); therefore, these variables were not accounted for in further analyses.

Intercorrelations between all variables included in the study are presented in Table 1. High levels of negative and positive thoughts (measured at T1 and T2) were associated with higher levels of ED symptoms. High levels of weight discrepancies were moderately correlated with high levels of ED symptoms. Further, high levels of positive, negative, and permissive thoughts were associated with high levels of the ED symptoms. Indices of weight discrepancies correlated positively with ED symptoms. Both types of weight discrepancies were strongly associated. For both types of weight discrepancies a similar pattern of associations with other study variables was observed.

### Testing the mediating role of automatic thoughts

Regarding the first hypothesis, mediating effects of automatic thoughts about eating in the relationship between weight discrepancies and ED symptoms were tested by two multiple mediation models (Table 2). Model 1 was designed to verify the indirect effects of negative (T2), positive (T2) and permissive thoughts (T2) (mediators) in the relationship between the actual (subjectively reported)–ideal weight discrepancy (T1) [the independent variable (IV)] and ED symptoms (T3) [the dependent variable (DV)]. Model 2 was verifying the indirect effects of all three types of thoughts (T2) (mediators) in relationship between the actual (objectively measured)–ideal weight discrepancy (T1) (IV) and ED symptoms (T3) (DV).

**Table 2:** Mediating effects of automatic thoughts about eating in the relationship between weight discrepancies and ED symptoms

Indirect effects pathways	*B*	SE	BC 95 % CI		*R* ^2^
			Lower	Higher	
Hypothesis 1: testing the mediating effects of automatic thoughts					
Model 1					
SI weight discrepancy T1 → negative thoughts T2 → ED symptoms T3	**0.032**	0.009	0.015	0.052	0.38
SI weight discrepancy T1 → positive thoughts T2 → ED symptoms T3	−0.002	0.000	−0.017	0.001	
SI weight discrepancy T1 → permissive thoughts T2 → ED symptoms T3	0.000	0.002	−0.003	0.010	
SI weight discrepancy T1 → negative thoughts T2 + positive thoughts T2 + permissive thoughts T2 → ED symptoms T3	**0.031**	0.009	0.015	0.052	
Model 2					
OI weight discrepancy T1 → negative thoughts T2 → ED symptoms T3	**0.033**	0.010	0.015	0.054	0.38
OI weight discrepancy T1 → positive thoughts T2 → ED symptoms T3	−0.002	0.004	−0.021	0.001	
OI discrepancy T1 → permissive thoughts T2 → ED symptoms T3	0.001	0.002	−0.002	0.013	
OI weight discrepancy T1 → negative thoughts T2 + positive thoughts T2 + permissive thoughts T2 → ED symptoms T3	**0.031**	0.010	0.015	0.050	

Values of indirect effect coefficient (*B*) presented in bold are significant. Each bootstrap was based on 1,000 repetitions. Bias corrected (BC) confidence intervals (CI) that do not include zero indicate a significant indirect effect

T1, Time 1; T2, Time 2; T3, Time 3; ED, eating disorders; SI weight discrepancy, actual (subjectively reported)–ideal weight discrepancy; OI weight discrepancy, actual (objectively measured)–ideal weight discrepancy

**Fig. 1:**
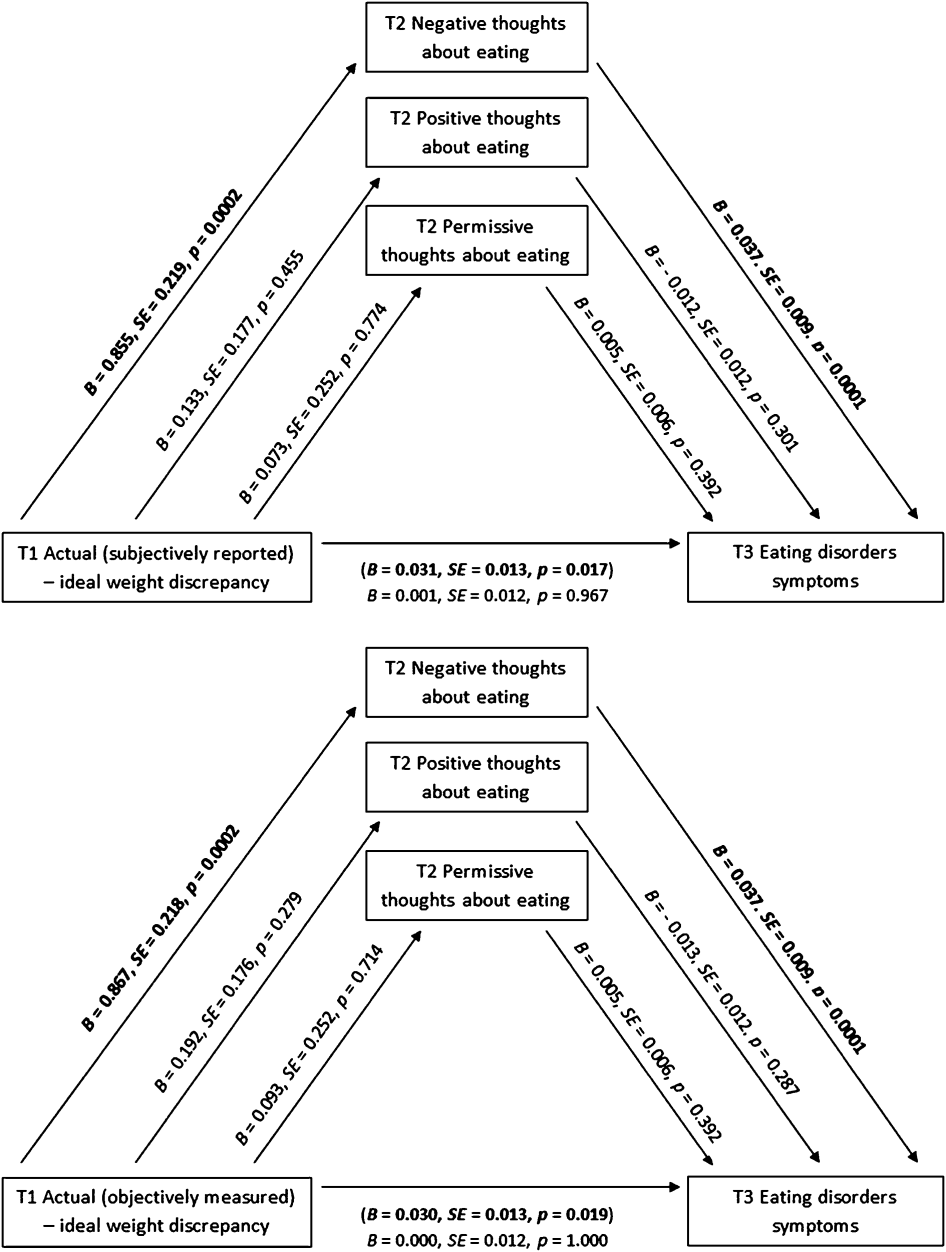
Thoughts about eating (T2) as mediators between the weight discrepancies (T1) and eating disorder symptoms (T3): results of the mediation analysis (significant coefficients are marked in bold)

The multiple mediation analyses in Model 1 showed that the association between the actual (subjectively reported)–ideal weight discrepancy (T1) and ED symptoms (T3) was mediated by negative thoughts (T2) as indicated by the significant indirect effect. Moreover, a total indirect effect for all three types of automatic thoughts (T2) emerged (see Model 1 in Table 2). This, however, was mainly driven by the indirect effect of negative thoughts, because the indirect effects of positive and permissive thoughts were not significant. Moreover, the associations between the IV and negative thoughts, and between negative thoughts and the DV were significant (Fig. 1, upper panel).

Similar results can be seen in Model 2 where negative thoughts (T2) and the three types of automatic thoughts together (T2) mediated the relationship between the actual (objectively measured)–ideal weight discrepancy (T1) and ED symptoms (T3) (see Model 2 in Table 2). Again, the total indirect effect is likely to be driven by the indirect effect of negative thoughts. Moreover, the associations between IV and negative thoughts, and between negative thoughts and DV were significant as well (Fig. 1, bottom panel). After entering the mediators in both models, the primary relation between the IV and DV became statistically insignificant and therefore the conservative conditions for mediation [18, 19] were met. In sum, the results confirm that the association between the weight discrepancies and the ED symptoms are mediated by negative but not by positive and permissive thoughts.

### Testing the mediating role of weight discrepancies

The second hypothesis, referring to the mediating effects of weight discrepancies in the relationship between automatic thoughts and ED symptoms, was tested with six mediation analyses (Models 3–8; Table 3). Single mediator models were conducted instead of models with multiple mediators, because the mediators were associated strongly. Models 3 and 4 were designed to verify the indirect effects of the actual (subjectively reported)–ideal weight discrepancy (T2) and the actual (objectively measured)–ideal weight discrepancy (T2) (mediators) in the relationship between the negative thoughts (T1) (IV) and ED symptoms (T3) (DV). Models 5 and 6 tested the indirect effects of both types of discrepancies (T2) (mediators) in relationship between the positive thoughts (T1) (IV) and ED symptoms (T3) (DV). Models 7 and 8 tested the indirect effects of those mediators (T2) in relationship between the permissive thoughts (T1) (IV) and DV (T3).

**Table 3:** Mediating effects of weight discrepancies in the relationship between automatic thoughts about eating and ED symptoms

Indirect effects pathways	*B*	SE	BC 95 % CI		*R* ^2^
			Lower	Higher	
Hypothesis 2: testing the mediating effects of weight discrepancies					
Model 3					
Negative thoughts T1 → SI weight discrepancy T2 → ED symptoms T3	**0.001**	0.0003	0.0002	0.0012	0.32
Model 4					
Negative thoughts T1 → OI weight discrepancy T2 → ED symptoms T3	**0.001**	0.0003	0.0001	0.0011	0.29
Model 5					
Positive thoughts T1 → SI weight discrepancy T2 → ED symptoms T3	0.002	0.002	−0.002	0.006	0.12
Model 6					
Positive thoughts T1 → OI weight discrepancy T2 → ED symptoms T3	0.002	0.002	−0.001	0.006	0.09
Model 7					
Permissive thoughts T1 → SI weight discrepancy T2 → ED symptoms T3	−0.002	0.003	−0.007	0.004	0.11
Model 8					
Permissive thoughts T1 → SI weight discrepancy T2 → ED symptoms T3	−0.001	0.002	−0.007	0.003	0.07

Values of indirect effect coefficient (*B*) presented in bold are significant. Each bootstrap was based on 1,000 repetitions. Bias corrected (BC) confidence intervals (CI) that do not include zero indicate a significant indirect effect

T1, Time 1; T2, Time 2; T3, Time 3; ED, eating disorders; SI weight discrepancy, actual (subjectively reported)–ideal weight discrepancy; OI weight discrepancy, actual (objectively measured)–ideal weight discrepancy

**Fig. 2:**
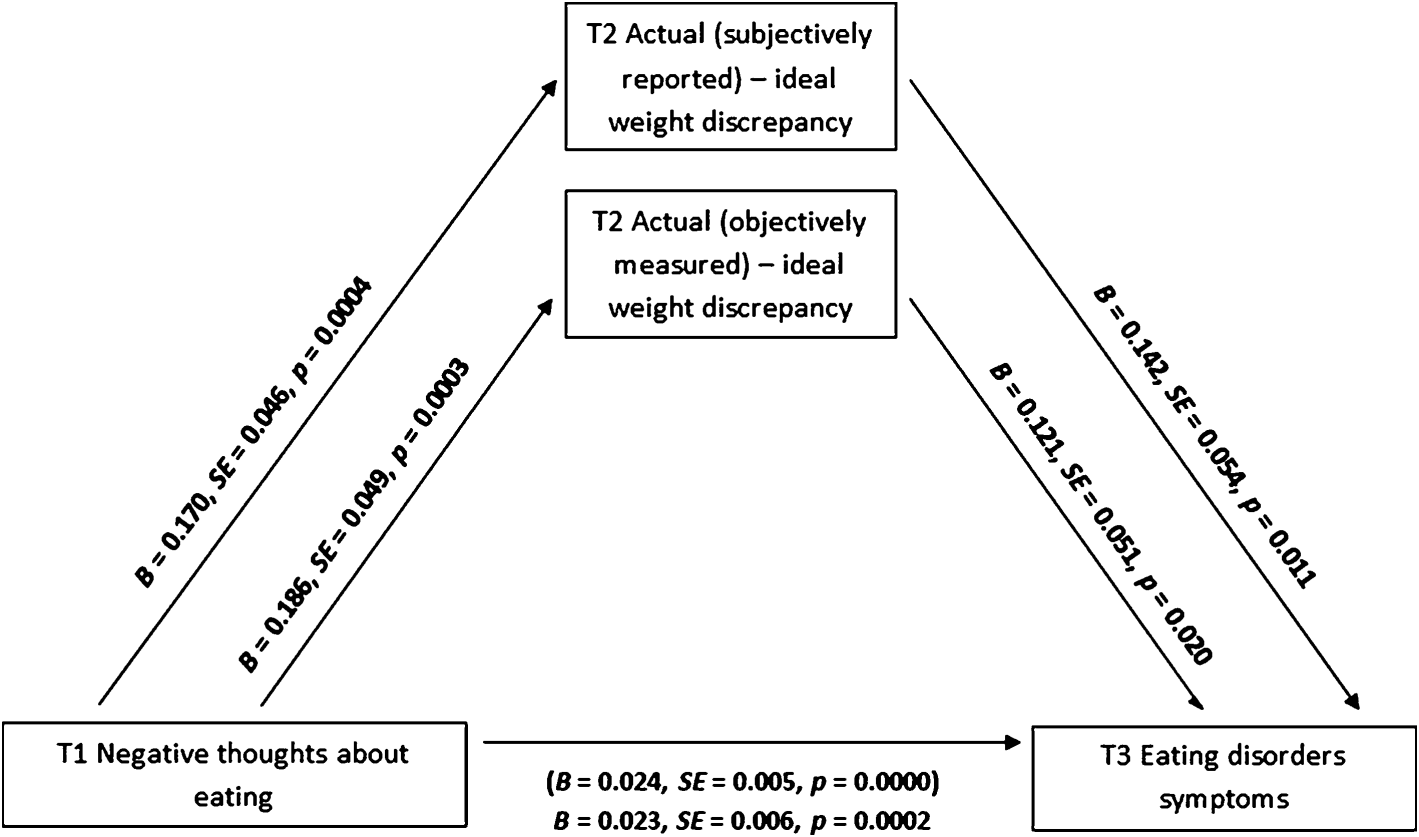
Weight discrepancies (T2) as mediators between negative thoughts about eating (T1) and eating disorder symptoms (T3): results of the mediation analysis

The mediation analyses in Models 3 and 4 showed that the associations between negative thoughts (T1) and ED symptoms (T3) were mediated by both types of weight discrepancies (T2) (see Models 3 and 4 in Table 3). The associations between IV and mediators, and between mediators and DV were significant (Fig. 2). The results of analyses conducted for other models indicated no significant indirect effects in association between positive and permissive thoughts (T1), weight discrepancies (T2), and ED symptoms (T3) (Table 3). In sum, both types of weight discrepancies are mediating the relationship between negative thoughts and ED symptoms.

## Discussion

This longitudinal study provided novel evidence for the association between cognitive factors, taking into account automatic thoughts about eating [1], weight discrepancies [10–13], and ED symptoms within a non-clinical sample of adolescents. In line with the cognitive ED models [1, 6] and in line with the self-discrepancy theory [10], it was assumed that these factors would play an important role in the process of formation and maintenance of ED symptoms. The results indicated that adolescents who reported high weight discrepancies (T1) were likely to present high levels of negative thoughts at T2, and consequently reported higher levels of ED symptoms at T3. In turn, adolescents who reported high levels of negative thoughts (T1) were likely to present high weight discrepancies at T2, and consequently reported higher levels of ED symptoms at T3. Both negative thoughts (T1) and weight discrepancies (T1) had significant indirect effects on ED symptoms evaluated at 11-month follow-up. Thus, weight discrepancies (T2) and negative thoughts (T2) acted as significant mediators.

These results are partly congruent with earlier research [2] indicating that women diagnosed with ED have more positive and negative thoughts about eating than dieting women (without the ED diagnosis) and a control group (non-dieting women without the ED diagnosis), whereas dieting women have higher levels of negative thoughts about eating than the control group. Our results showed that the effects of positive thoughts were negligible, whereas we found mediating effects of negative thoughts. As indicated in earlier research [2], the presence of positive thoughts is characteristic for individuals diagnosed with ED compared to those who do not meet diagnostic thresholds. Thus, limited effects of positive thoughts in this study may be associated with the specificity of the present sample, which was a non-clinical group of adolescents who presented mild ED symptoms.

The results are also consistent with previous research [21], showing that a low awareness of body appearance among adolescents increases the discrepancies between their bodies (actual-self) and the ideal body shape (ideal-self), as well as between their bodies and the image of how the body of a person of a certain age should look to be socially accepted and attractive. These discrepancies may lead to the formation of ED symptoms. Our findings add novel evidence explaining the underlying processes of ED symptoms’ development in a non-clinical population.

Our research provides a novel insight into the reciprocal processes between automatic thoughts and weight discrepancies, predicting formation of ED symptoms in non-clinical samples. The findings indicate the reciprocal relationships between two predictors of ED symptoms: negative thoughts and the actual–ideal weight discrepancies. A higher level of either negative thoughts or weight discrepancies was related to an increased level of the respective other predictor at the later measurement point. Thus, we found that negative thoughts and the weight discrepancies constitute a vicious cycle, related to higher ED symptoms. The results of the present study indicated that both types of actual–ideal weight discrepancies (objectively measured and subjectively reported) were very strongly associated. The use of either measure of discrepancies resulted in almost identical patterns of associations with automatic thoughts and ED symptoms. Previous research showed that the differences between self-reported and objectively measured body weight are usually negligible [22]. Our findings add to existing knowledge showing that ideal–actual weight discrepancies play similar roles, regardless the use of objective or subjective body weight measure.

Some limitations must be taken into account as well. The first concerns the size of the study sample, which could have affected the statistical power of the analyses. The results may be also affected by the specificity of the group, i.e. a non-clinical group of adolescents. The aim of this study, however, was to provide insight into potential mechanisms of developing ED. Thus, a clinical population with manifest ED would not have been appropriate to answer this research question. Moreover, it is possible that social desirability affected the participants’ responses, but its effects were not controlled for. The SCOFF Questionnaire used in the study is a screening tool for identifying ED symptoms. Therefore, the results do not provide enough information about the associations among negative thoughts, the actual–ideal weight discrepancies, and ED symptoms specific for different types of ED (e.g. anorexia nervosa, bulimia nervosa, binge eating). Using tools measuring complex symptoms of anorexia and bulimia in future studies may allow evaluating distinct effects of thoughts and discrepancies in these two disorders. Finally, other cognitive models of ED, emphasizing the role or self-esteem, clinical perfectionism, and mood intolerance [7, 9] provide competing assumptions regarding the formation and maintenance of ED symptoms, in particular when individuals with bulimia and anorexia are analyzed separately. Thus, future studies might want to competitively compare the model used in our study to other cognitive models of ED to advance our knowledge with regard to the development—and potential prevention—of ED.

Results of this study indicate that thoughts about eating should be taken into account when considering ED prevention in the general population or in screening for adolescents at risk for developing ED symptoms. Our findings suggest that they are associated with the occurrence of ED symptoms and explain the relation between the weight discrepancies and the ED symptoms. The actual–ideal weight discrepancies and thoughts about eating form a vicious cycle which may lead to formation of ED symptoms among adolescents. Particular attention should be paid to adolescents who manifest large weight discrepancies and report high prevalence of negative thoughts about eating as they may be at the highest risk of developing ED symptoms.
